# ”Who’s Calling?” Challenges and Strategies for Recruitment of Assisted Living Communities in Research Studies

**DOI:** 10.1093/geroni/igaf122.3193

**Published:** 2025-12-31

**Authors:** Debra Dobbs, Jonathan Clapp, Hongdao Meng, Lindsay Peterson, Layla Katharine Santana, William Haley

**Affiliations:** University of South Florida, Tampa, Florida, United States; University of South Florida, Tampa, Florida, United States; University of South Florida, Tampa, Florida, United States; University of South Florida, Tampa, Florida, United States; University of South Florida, Tampa, Florida, United States; University of South Florida, University of South Florida, Florida, United States

## Abstract

The Assisted Living and Residential Care (AL/RC) industry is a complex, state regulatory model of care has been of keen interest to researchers for the last 25 years. Of particular note is the fact there has been a cadre of researchers in the U.S. that have been part of the Center for Excellence in Assisted Living (CEAL, now CEAL@UNC) since the early 2000s invested in addressing the care quality of residents who receive and staff who provide care. Because AL/RC communities vary by state in their regulatory structures and policies, and there is frequent turnover of ownership and administrative staff, recruitment of AL/RCs can be challenging for researchers. This study will detail AL/RC recruitment strategies for a National Institute on Aging funded clinical trial to implement the Palliative Care Education in Assisted Living for Dementia Care Providers (PCEAL-DCP). Effective strategies for recruitment have included: 1) developing a community/university partnership with key current and previous AL owners and operators who can assist with outreach to their networks; 2) building a consortium of AL communities from prior study participation; 3) direct in-person and social media inquiries versus costly and timely traditional recruitment of mail and telephone calls; 4) making the business case for the value of participation in research to corporate AL chains; and 5) coordinating with hospice community partners who provide continuing education incentives and existing relationships with AL community providers. Study design in terms of budgetary and timeline issues to consider in recruitment of ALs will also be discussed.

